# Attacks of *Kalotermes flavicollis* Fabricius (1793) and Associated *Aspergillus* Micheli ex Haller (1768) Species

**DOI:** 10.3390/insects15110899

**Published:** 2024-11-18

**Authors:** Giulia Mirabile, Livio Torta, Marika Lamendola, Maria Concetta Rizzo, Barbara Manachini

**Affiliations:** 1 Dipartimento di Scienze Agrarie, Alimentari e Forestali (SAAF), Università degli Studi di Palermo, Viale delle Scienze, 90128 Palermo, Italy; giumirabile@gmail.com (G.M.); marika.lamendola@unipa.it (M.L.); mariaconcetta.rizzo@unipa.it (M.C.R.); barbara.manachini@unipa.it (B.M.); 2 Ispettorato Provinciale dell’Agricoltura di Palermo—Regione Sicilia, Via Camillo Camilliani 54, 90145 Palermo, Italy

**Keywords:** termite infestations, entomopathogenic fungi, new insect–fungi association, insect–fungi ecological association

## Abstract

The yellow-necked dry-wood termite *Kalotermes flavicollis* (Fabricius, 1793) (Blattodea: Kalotermitidae) is an important pest that infests wood in Europe. An increase in attacks by *K. flavicollis* has been seen in buildings and in churches in Palermo (Italy), with attacks on both structural elements and artefacts. Future climate changes are expected to lead to increasing temperatures, which will probably affect the pest status of this species, which is difficult to control. In this context, it is important to identify potential natural antagonists of *K. flavicollis*. During a survey of the *K. flavicollis* population, several dead individuals with evident fungal efflorescence were found. Therefore, a study aimed at the isolation and identification of these microorganisms was conducted. Fungal colonies isolated from mycelial structures grown on insects were identified based on morphological characteristics and DNA profiling. Three different species were identified: *Aspergillus nomius*, *A. subramanianii*, and *A. tamarii*. This is the first time that fungi have been recorded in association with the yellow-necked dry-wood termite and, in addition, this study reports the first association of *A. subramanianii* with Isoptera.

## Simple Summary

The yellow-necked dry-wood termite, *Kalotermes flavicollis*, is a significant pest in wooden structures in Europe, particularly churches and museums. An increase in infestations has been observed in Palermo, Italy, affecting both structural elements and artefacts. A study aimed at gaining further knowledge of this insect permitted the isolation and identification of three fungal species associated with dead termites: *Aspergillus nomius*, *A. subramanianii,* and *A. tamarii*. This is the first report of fungi linked to *K. flavicollis* and the first recorded association of *A. subramanianii* with Isoptera (order of social insects that live in colonies, including termites). Further research will be conducted to define the possible ecological relationships between these organisms.

## 1. Introduction

*Kalotermes flavicollis* (Fabricius, 1793) (Blattodea: Kalotermitidae), known as the ‘yellow-necked dry-wood termite’, mainly lives in regions of the Mediterranean basin, infesting broad-leaved trees and, therefore, having a certain economic impact in the agricultural and forestry fields [1,2,3,4]. In many regions of the world, termites are considered a serious problem as they can cause structural damage that results in significant financial losses [5]. Economically significant damage caused by *K. flavicollis* has been recorded in vineyards, orchards, and urban trees [6,7]. *K. flavicollis*, is capable of attacking wood and nesting inside it as well as the ground outside. This termite is widespread in urban environments, where it colonizes wooden artefacts and structural elements of buildings, threatening cultural heritage elements and damaging structures of significant artistic value [8,9,10]. Optimal climatic conditions for termites include high temperatures and a low rate of precipitation. The heating and drying of climates that global warming is causing in many areas of the planet make them more active and faster in devouring wood. A termite colony at 30 °C eats wood seven times faster than one active at 20 °C (https://www.naturalmentescienza.it/sections/?s=4146, accessed on 8 November 2024).

Termite control involves the use of various methods to prevent or manage termite infestations, which are broadly categorized as physical, biological, and chemical methods [11]. However, controlling termites, including *K. flavicollis*, is particularly difficult, especially in the context of cultural heritage. Several products based on botanical extracts [12] as well as chemical products, such as juvenile hormone analogues [13], have been tested against termites with contrasting results, depending on the species and its biology. Chemical means involve the use of insecticides to kill or repel termites. Chlorinated hydrocarbons, such as chlordane and lindane, were commonly used in the past but are now banned due to their persistence in the environment and potential health risks. Additionally, other insecticides that were used in termite bait, such as diflubenzuron, part of the benzoylurea class, or hexaflumuron, a chitin synthesis inhibitor that causes termites to die when they moult [14,15], are now or banned or restricted for specific uses in Europe and other countries [16,17,18]. For these reasons, biological means of termite control involving natural enemies or termite pathogens to manage their populations are a pillar of the Integrated Pest Management protocol against termites.

Biocontrol strategies based on the use of microbial entomopathogenic agents, in particular *Metarhizium anisopliae*, a fungus tested with some success in vitro, seem to represent a promising control alternative for various termite species [19]. In effect, biocontrol of termites is an area of ongoing research, which could become a control strategy.

A recent study based on Springhetti’s historical collection of termites [20] highlighted the geographical distribution of *K. flavicollis*, also reporting that this species is the most common termite species in Sicily [21]. However, *K. flavicollis* was recorded mainly in orchards, especially in vineyards, carob, and olive trees, in both coastal and inland areas.

In this study, the termite’s presence was recorded primarily in buildings, and it was ascertained by monitoring various locations in the municipality of Palermo (Italy) from 2019 to 2021. The lucifugal behaviour of this insect, together with the lockdown period following the COVID-19 health emergency, hindered early identification of infestations. During the monitoring, some dead, winged individuals with evident associated fungal structures were detected and the fungal microorganisms were isolated and identified. The ecological behaviour of the identified fungi could provide indications as to the nature of the insect–fungi relationship.

## 2. Materials and Methods

### 2.1. Sampling and Identification

During the three-year period of monthly monitoring from 2019 to 2021, several termite nests were found in Palermo (Italy). The adult insects were collected from their mother colonies in October, during their flight period, and then maintained at 4 °C until use in laboratory tests. Furthermore, wherever possible, pseudoergates and soldiers were also captured from some portions of the nest. Winged individuals of the yellow-necked dry-wood termites were identified based on the description in Springhetti [22] and their characteristic barrel-shaped feces [23]

### 2.2. Isolation and Morphological Identification of Fungal Colonies

Alive *K. flavicollis* specimens were collected and brought into the laboratory, where they were subjected to laboratory assays for the isolation of fungal colonies. In particular, on 14 individuals with evident fungal efflorescence, direct (collection of mycelial masses) and indirect (serial dilutions) isolation techniques were applied using the agarized medium PDA (Potato Dextrose Agar, Oxoid, Waltham, MA, USA), testing 7 insects with each technique. In the first case, insects were observed under a stereoscopic microscope, under a hood and near a flame.

With the aid of a sterile needle, portions of the fungal efflorescence were taken from the body of the insects and placed directly into Petri dishes (Ø 10 cm; 5 portions per plate, 3 plates per insect). In the second case, each individual was put into a test tube containing 10 mL of sterile distilled water and, after vortexing, 1 mL of the suspension was diluted in 9 mL of sterile distilled water in a new test tube. The suspension was thus diluted four times, reaching a concentration of 10–4. For each suspension, 3 aliquots of 100 µL were taken and each aliquot was distributed in a Petri dish and appropriately spread on the surface of the PDA. All the inoculated plates were incubated at 24 °C in the dark and observed daily for 10 days in order to detect the growth of fungal colonies. At the end of the incubation period, the developed colonies were transferred to PDA, and the pure colonies were used for morphological and molecular analysis.

Small portions of mycelial mass grown in PDA plates were mounted with a drop of lactophenol solution (25 mL distilled water, 25 mL glycerin, 25 mL lactic acid, 25 g phenol crystals), adding 0.01% methylene blue. Microscopic observations aimed at identifying the genus were conducted using a light microscope (Axioskop; Zeiss, Oberkochen, Germany) coupled with an AxioCam MRc5 (Zeiss, Oberkochen, Germany) digital camera. Images were captured using the AxioVision 4.6 software (Zeiss, Oberkochen, Germany). All obtained fungal colonies were grouped into morphotypes according to their macroscopic and microscopic features [24].

### 2.3. Molecular Identification and Phylogenetic Analysis of the Isolated Fungi

One strain for each *Aspergillus* group was selected for DNA extraction. DNA was extracted from the mycelium of 7-year-old pure colonies grown on PDA using the Extract-N-Amp™ extraction kit (Sigma-Aldrich, St. Louis, MI, USA) following the manufacturer’s instructions. The internal transcribed spacer region (ITS) of rDNA was amplified by PCR using the universal primers ITS1F and ITS4. The reaction was performed in a total volume of 20 µL, consisting of the following: 10 µL the Extract-N-Amp PCR reaction mix (Sigma-Aldrich, St. Louis, MI, USA), 4 µL of sterilized distilled water, 1 µL of each primer (10 µM), and 4 µL of extracted DNA. The amplification was carried out in a MultiGene OptiMax thermocycler (Labnet International Inc., Edison, NJ, USA) as follows: an initial denaturation cycle at 94 °C for 3 min; 35 cycles at 94 °C for 30 s; annealing at 55 °C for 30 s; elongation at 72 °C for 45 s; and a final extension at 72 °C for 10 min. PCR products were separated by electrophoresis in 1.5% agarose gel and detected under UV transilluminator. PCR products were sent to BMR Genomics (Padova, Italy) for purification and sequencing.

The obtained sequences were compared with those of the GenBank database using BLASTn (https://blast.ncbi.nlm.nih.gov, accessed on 15 May 2022), manually adjusted when needed and deposited on GenBank to obtain the Accession numbers. An ITS-phylogenetic analysis was performed using MEGA11. Our sequences were aligned with representative *Aspergillus* sequences retrieved from NCBI using ClustalW software 1.81. A Neighbour-Joining starting tree was automatically generated by MEGA11 and 1000 bootstrap replicates were performed.

## 3. Results

### 3.1. Termite Identification and Fungal Apparence

**Figure 1:**
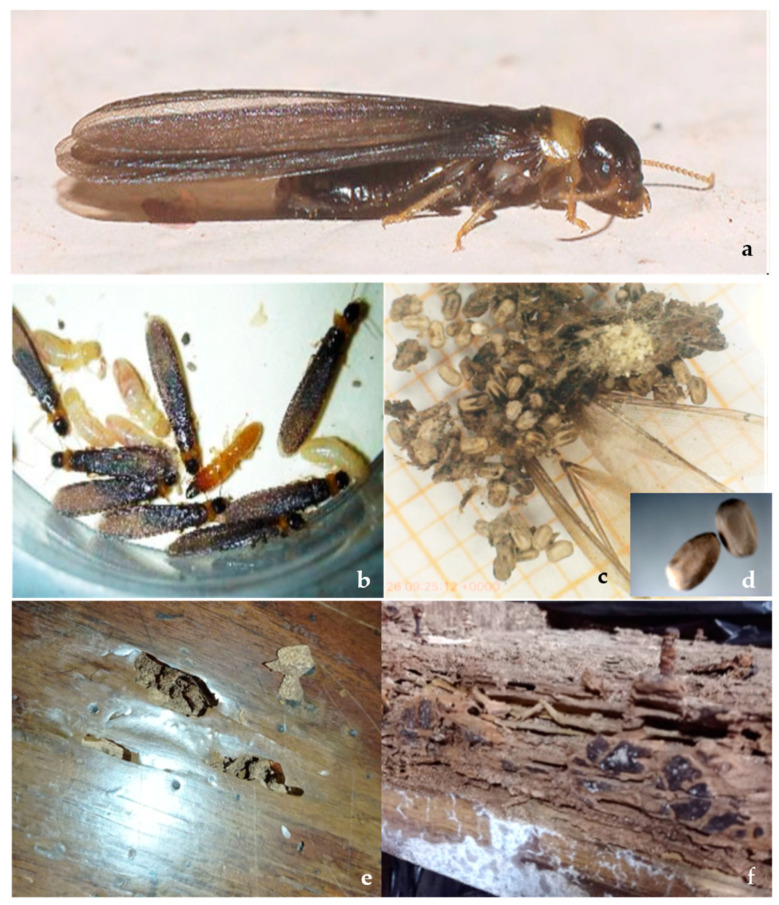
(**a**) *Kalotermes flavicollis* Fabr. (Blattodea: Kalotermitidae); (**b**) pseudoergates and soldiers; (**c**,**d**) characteristic barrel-shaped feces of *K. flavicollis*; (**e**) termite nets in dry and (**f**) humid wooden materials.

**Figure 2:**
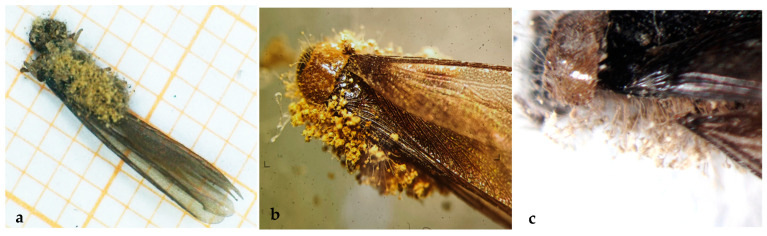
(**a**–**c**): fungal structures emerging from insects observed by stereoscopic microscope.

The specimens collected were identified according to Springhetti [22], as adults of yellow-necked dry-wood termites *Kalotermes flavicollis* sensu stricto of lineage A [3] (Figure 1a). Pseudoergates and soldiers were also captured from some portions of the nest (Figure 1b). Their characteristic barrel-shaped feces [23] (Figure 1c,d), also confirme the identification, as *K. flavicollis* sensu stricto of lineage A [3]. Nests were found located in dry (Figure 1e) and humid wooden materials (Figure 1f). Fungal growth and fungi emerging from the insect’s body to produce spores were observed on insect cadavers both in the specimens collected in the field and in the ones brought alive, dead in average in 7 days, in the laboratory (Figure 2a–c). 

### 3.2. Isolation and Morphological Identification of Fungal Colonies

**Figure 3:**
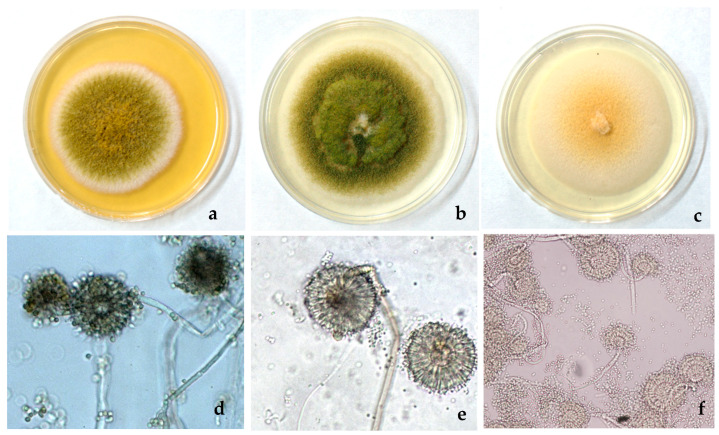
Colonies: (**a**) olive in colour with white margins; (**b**) greyish-green–brown; (**c**) white granular margin and yellow centre. (**d**) *Aspergillus tamarii*; (**e**) *A. nomius*; (**f**) *A. subramamanianii*.

For both isolation techniques, fungal colonies grew from the third day after incubation. By the use of the direct isolation technique, 76 fungal colonies were obtained, with most of them (90%) showing a floccose texture. In particular, 50% of the total colonies were olive in colour with white margins (Figure 3a), 30% had a greyish-green–brown colour with faster radial growth (Figure 3b), and some of them (20%) had a yellow centre with white granular margins and sclerotia on the surface, and were light yellow in colour on their reverse side (Figure 3c).

A total of 183 colonies with similar features to those described above, and with similar distribution percentages, were isolated using the serial dilution technique. Observed under stereoscopic microscope, in all colonies, conidial heads were highlighted, and microscopic observation confirmed that all of them belonged to the genus *Aspergillus* (Figure 3d–f). From each of the three morphotype groups, one isolate was selected, named SAAF I2, SAAF I6, and SAAF I9, respectively, and submitted for molecular identification.

### 3.3. Molecular Identification and Phylogenetic Analysis of the Isolated Fungi

**Figure 4:**
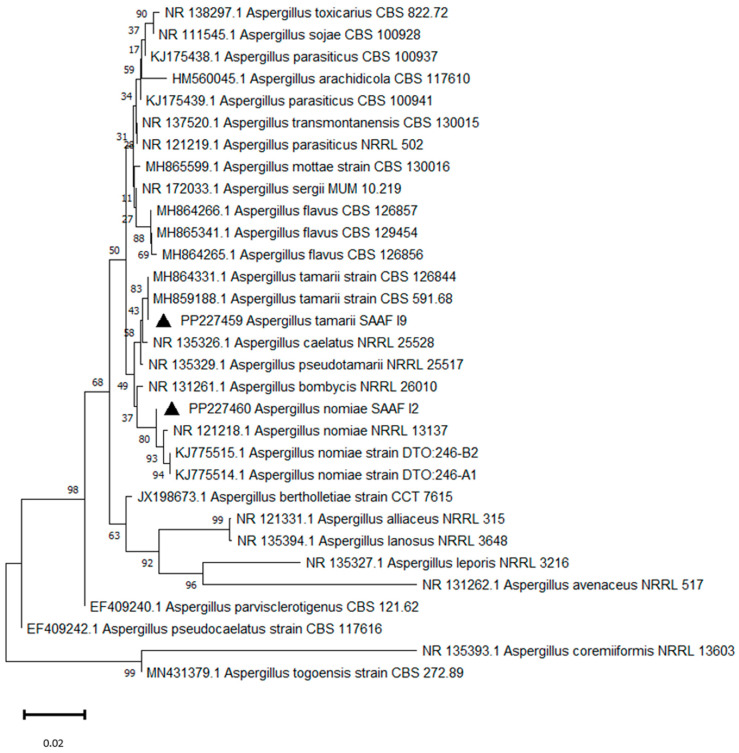
Neighbour-Joining tree based on ITS sequence analysis of *Aspergillus* section *Flavi*. The isolates obtained in this study and deposited in the GenBank database are indicated with black triangles. Bootstrap percentages are indicated at nodes; 1000 replications were performed.

**Figure 5:**
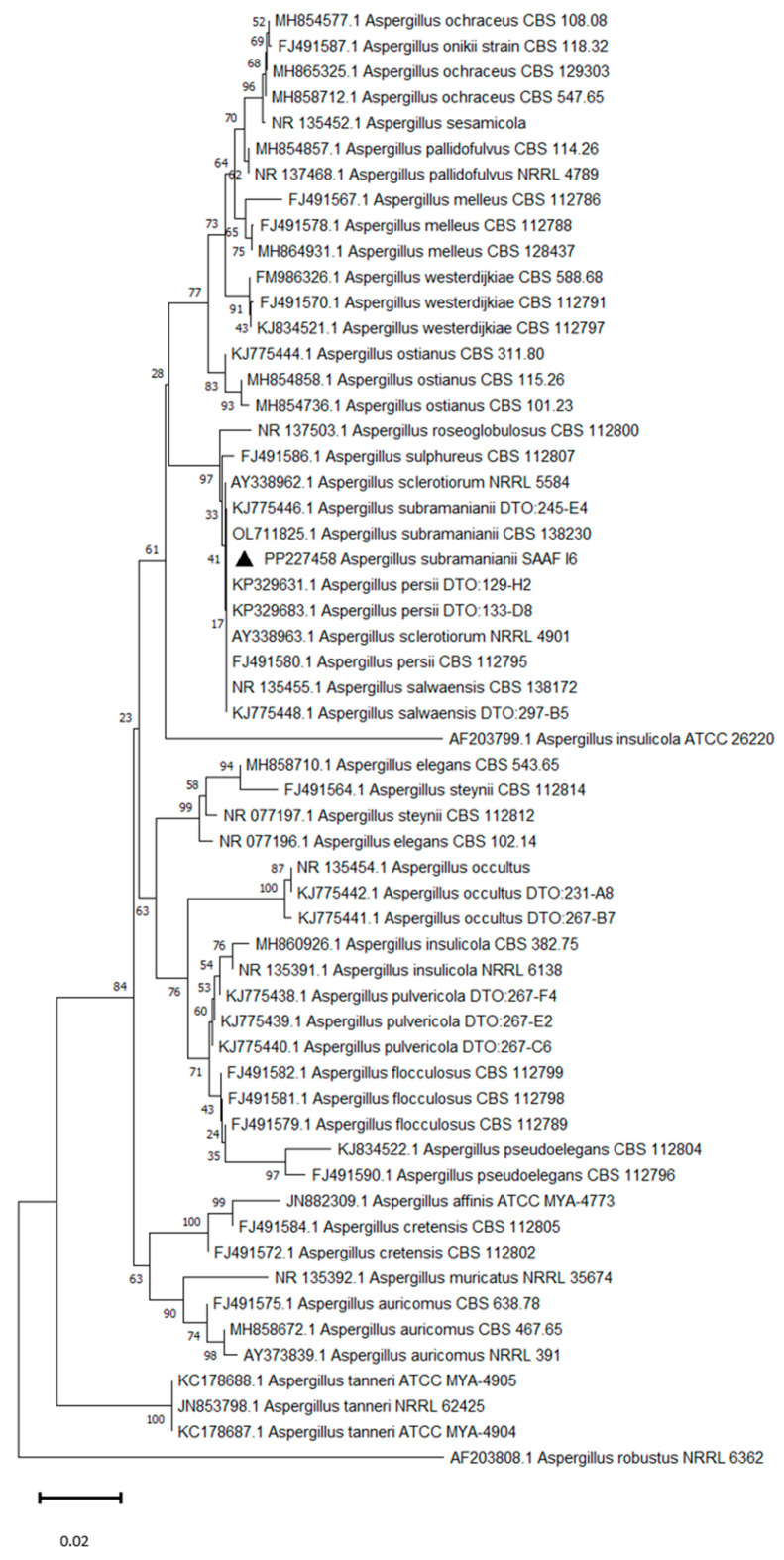
Neighbour-Joining tree based on ITS sequence analysis of *Aspergillus* section *Circumdati*. The isolates obtained in this study and deposited in the GenBank database are indicated with black triangles. Bootstrap percentages are indicated at nodes; 1000 replications were performed.

Phylogenetic analysis based on ITS sequences of *Aspergillus* section *Flavi* and *Aspergillus* section *Circumdati* showed that the SAAF 12, SAAF 16, and SAAF 19 isolates grouped with *A. nomius* Kurtzman, B.W. Horn & Hesselt, *A. subramanianii* Visagie, Frisvad & Samson, and *A. tamarii* Kita, respectively, with high bootstrap values (Figure 4 and Figure 5).

## 4. Discussion

The determination of the presence of *K. flavicollis* in the Municipality of Palermo confirms its already reported spread along the Mediterranean coasts of Europe, Africa, and Asia, and the monitoring results support the information already known regarding its ecology and harmfulness.

The emergence of winged adults and the consequent swarming of *K. flavicollis* that occurred in autumn confirms previous observations in southern Italy [21]. During the collection process, no colour variations in *K. flavicollis* populations were recorded; in the past, alates with a pronotum of a darker shade of yellow, a dark posterior margin, or which were even entirely dark, of the same colour as the head, were reported for some Italian localities, including Sicily, and were considered a different species, *K. italicus* Ghesini and Marini, 2013 [25].

Regarding fungal microorganisms associated with termites, there are data in the literature for the species *Coptotermes formosanus* Shiraki 1909 [26], *Psammotermes hybostoma* Desneux, 1902 [27,28], and several species of *Reticulitermes* Holmgren, 1913 [29]. In particular, *A. nomius* has been reported on *C. formosanus* individuals subjected to some kind of stress [26], both as a saprophyte and as a facultative parasite, while *A. tamarii* has been identified in association with *P. hybostoma* [27].

This study reports the first *A. subramanianii* association with termites. To our knowledge, this record is also the first report of fungal microorganisms associated with *K. flavicollis*. However, there are no certain data regarding the role that these fungal organisms play in relation to termites. [30]. However, among the numerous forms of ecological association between insects and fungi, trophic or neutral relationships cannot be excluded, given the ecological needs common to both organisms [31]. In fact, fungi of the *Aspergillus* genus, as well as termites, prefer hot, humid conditions and environments with poor ventilation, such as those inside the insect nests. Furthermore, all termites and many species of *Aspergillus* can use cellulose as a trophic source. Future investigations relating to the ecological role of these fungi, both individually and in association and their possible interactions with *K. flavicollis* colonies, might provide useful indications, especially with a view to the development of targeted biocontrol strategies.

## Data Availability

Data will be provided upon reasonable request by Barbara Manachini and Livio Torta.
